# Improved Resolution and Cost Performance of Low-Cost MEMS Seismic Sensor through Parallel Acquisition

**DOI:** 10.3390/s21237970

**Published:** 2021-11-29

**Authors:** Xing-Xing Hu, Xi-Zhen Wang, Bo Chen, Cai-Hua Li, Yi-Xiang Tang, Xiao-Yu Shen, Yuan Zhong, Zhuo-Lin Chen, Yun-Tian Teng

**Affiliations:** 1 Institute of Geophysics, China Earthquake Administration, Beijing 100081, China; huxx@cea-igp.ac.cn (X.-X.H.); WXZ@cea-igp.ac.cn (X.-Z.W.); chenBo@cea-igp.ac.cn (B.C.); lich@cea-igp.ac.cn (C.-H.L.); tangyx@cea-igp.ac.cn (Y.-X.T.); shenxy@cea-igp.ac.cn (X.-Y.S.); zhongy@cea-igp.ac.cn (Y.Z.); ChenZL@cea-igp.ac.cn (Z.-L.C.); 2 Institute of Disaster Prevention, Sanhe 065201, China

**Keywords:** earthquake intensity reporting, earthquake early warning, MEMS accelerometer, correlation average, cost performance

## Abstract

In earthquake monitoring, an important aspect of the operational effect of earthquake intensity rapid reporting and earthquake early warning networks depends on the density and performance of the deployed seismic sensors. To improve the resolution of seismic sensors as much as possible while keeping costs low, in this article the use of multiple low-cost and low-resolution digital MEMS accelerometers is proposed to increase the resolution through the correlation average method. In addition, a cost-effective MEMS seismic sensor is developed. With ARM and Linux embedded computer technology, this instrument can cyclically store the continuous collected data on a built-in large-capacity SD card for approximately 12 months. With its real-time seismic data processing algorithm, this instrument is able to automatically identify seismic events and calculate ground motion parameters. Moreover, the instrument is easy to install in a variety of ground or building conditions. The results show that the RMS noise of the instrument is reduced from 0.096 cm/s^2^ with a single MEMS accelerometer to 0.034 cm/s^2^ in a bandwidth of 0.1–20 Hz by using the correlation average method of eight low-cost MEMS accelerometers. The dynamic range reaches more than 90 dB, the amplitude–frequency response of its input and output within −3 dB is DC −80 Hz, and the linearity is better than 0.47%. In the records from our instrument, earthquakes with magnitudes between M2.2 and M5.1 and distances from the epicenter shorter than 200 km have a relatively high SNR, and are more visible than they were prior to the joint averaging.

## 1. Introduction

Earthquakes are common natural phenomena on Earth. According to statistics, approximately 1 million earthquakes occur every year [1], and approximately 1000 of them can cause damage. To reduce the losses caused by earthquakes to human beings, long-term monitoring of earthquakes, especially destructive medium-strong earthquakes, has become an important means of earthquake prevention, mitigation, and research. While there are currently no reliable methods to accurately predict earthquakes, seismic instruments can rapidly detect an earthquake as it begins to unfold and allow those monitoring the data to communicate a warning to the community prior to the onset of ground shaking. In the absence of earthquake prediction techniques, countries and regions that experience frequent earthquakes often rely on earthquake early warning systems [2,3,4] and post-earthquake seismic intensity rapid reporting tools [5] to minimize human losses and property damage [3,6,7]. In the last decade, earthquake early warning systems have evolved rapidly in terms of their theoretical and methodological advances [2,5,7,8]. For example, point source algorithm, finite fault, and ground motion models have been developed in the methods applied to EEW [2]. A new generation of algorithms has emerged. The propagation of the local undamped motion (PLUM) algorithm [9] has undergone substantial refinement and testing in Japan [2]. Mexico, Japan, South Korea, and Taiwan have all established earthquake early warning systems (EEW systems) [10,11,12,13,14,15,16,17,18,19,20,21,22]; Turkey, Romania, Italy, China, Israel, and southwestern Iberia are on the verge of developing and testing their own seismic intensity rapid reporting systems [10,14,23,24,25,26,27,28]. The working principle of an EEW system is usually based on the idea that P-waves are faster than destructive S-waves. A close-to-earthquake seismic sensor means that the estimated earthquake location and magnitude can be obtained soon after the arrival of P-waves, and the EEW system can serve farther warning target areas [4,29]. Seismic intensity rapid reporting is the process of recording, analyzing, and reporting the seismic intensity through the real-time monitoring of data from existing stations and data from rapidly deployed instruments located near the quake-prone area. Compared to quantifying the seismic influence field using post-earthquake artificial field surveys, seismic intensity rapid reporting provides near-real-time estimations of the seismic impact (using the recorded intensity and the ground motion parameters) of each observation point, empowering authorities to make quick decisions about earthquake emergency rescue scenarios, minimizing casualties, and mitigating economic loss. With a higher seismic monitoring station density, it is possible to have a more comprehensive understanding of the seismic influence field [3,30]. Compared with the traditional artificial seismic intensity field investigation, seismic instrument intensity rapid reporting has the characteristics of objectivity, timeliness, and indirect response to earthquake damage. However, it will significantly increase the expense of EEW systems to build a dense seismic monitoring network with the traditional strong motion seismographs [4].

Therefore, the performance of an EEW system is greatly determined by the density of the EEW network [4]. Because intensive observation is a remarkable feature of earthquake early warning and earthquake intensity rapid reporting systems [31], the observation cost of each monitoring point should be reduced as much as possible to reduce the cost of the network system [32]. To reduce instrument costs and establish high-density monitoring networks, a new type of low-cost accelerometer based on the Micro-Electro Mechanical System (MEMS) was introduced to seismic applications beginning in the 1990s [33]. Although the low-cost MEMS accelerometer has a lower resolution compared with the electromechanical sensors of traditional seismographs, it has extremely low cost, negligible volume, small power consumption, and wide frequency band (for example, for ST company’s LIS3DHH three-axis digital output MEMS accelerometer, noise is 45 ug/√Hz, frequency band width is 235 Hz or 440 Hz, and price is USD 7.84 within 500 pieces, according to the website of the ST, 16 November 2021). MEMS accelerometers have quickly been adopted for wide use in earthquake early warning and seismic intensity rapid reporting systems and other medium-strong earthquake monitoring approaches [34,35]. In 2010, the Earthquake Early Warning Research Group of National Taiwan University (NTU) developed a P-alert device based on MEMS accelerometers and formed an EEW test network system in June 2012 [36]. They used a low-cost MEMS accelerometer to develop a seismic intensity rapid reporting instrument that can output ground motion parameters in real time and that can quickly measure the vibration intensity within a few seconds to tens of seconds after the earthquake occurs [36]. Fu et al. [4] used an ADXL355 accelerometer to develop a low-cost Class C MEMS sensor for EEW high-density networks, with a dynamic range of 87 dB, capable of detecting small earthquakes from M3.1 to M3.6 within 50 km [4].

**Figure 1:**
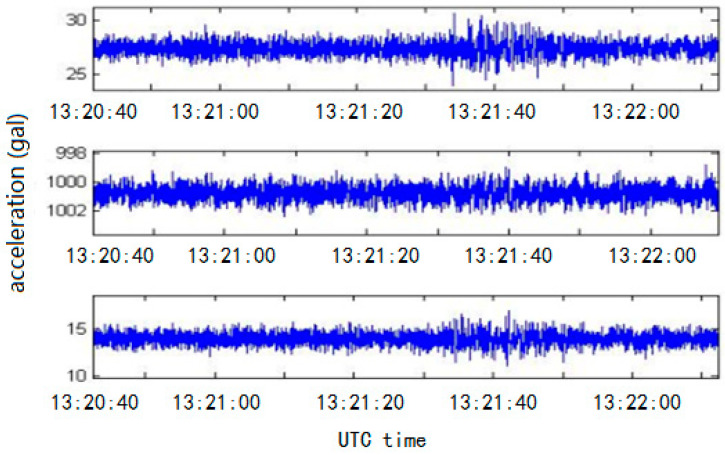
Three-component seismic waveform data recorded by the low-precision MEMS seismic intensity meter. (The event is UTC2017-8-8 13:19:46 Jiuzhaigou M7.0 earthquake in Sichuan, China. The hypocentral depth is 20 km. The monitoring network is 360 km away from the epicenter. The model of MEMS acceleration sensor used is MMA8451Q, and the sampling rate of the seismic sensor is 50 Hz.)

These low-cost commercial MEMS digital accelerometers have a number of advantages: they are small, record data in three directions, consume little power, and do not require an AD conversion circuit or calibration. However, these accelerometers record data at a relatively low resolution; as a result, signal and instrument noise can interfere with weak seismic signals, leading to inaccuracies in the estimation of the early warning seismic parameters [10,34]. While a low-cost MEMS digital accelerometer can record the maximum amplitude of large earthquakes and calculate the corresponding ground motion parameters (such as PGA and PGV), the signal-to-noise ratio (SNR) is not high enough to successfully resolve the P-wave arrivals of small or distant earthquakes (Figure 1), and the instrument has a few functions, which limits the widespread application of these instruments, especially as part of an earthquake early warning network. To improve the performance of the EEW system, Peng et al. [32] used three MT Microsystems Co., Ltd.’s (Shijiazhuang, Hebei, China) MSV6000-02 accelerometers and a 24-bit TI’s ADS1281 analog-to-digital convertor (ADC) to develop a Class B sensor with a dynamic range of 98 dB [32]. Such high-precision MEMS accelerometers are often analog output and single-component, difficult to integrate, and require more complex AD conversion. The entire mechanism of an intensity rapid reporting instrument must be calibrated carefully, and the cost of the accelerometer (hundreds of USD) is much higher than that of a three-component integrated MEMS accelerometer with lower accuracy (a few USD). Therefore, the cost of raw materials and manual adjustment for an integrated instrument is much higher than that of a seismic sensor comprising a three-component MEMS digital accelerometer.

In this article, we propose the use of multiple low-cost and low-resolution digital MEMS accelerometers to improve the resolution of small signals by means of a correlation average. We developed a seismic sensor with high-resolution and low-cost that uses a low-power high-performance ARM embedded processing system and a Linux operating system to create a low-power data acquisition system. With a built-in 32 GB SD memory card, the instrument can continuously store real-time data for approximately 12 months at a sampling rate of 50 SPS. With the ability to transmit data over a wired IP network and apply a processing algorithm to real-time seismic data [37], it is able to automatically calculate ground motion parameters such as PGA and PGV [38]. With its low cost, low power consumption, small size, and processing ability, this small digital instrument can be easily deployed in a seismic intensity rapid reporting or earthquake early warning network. It can also be used to monitor landslides [39,40] and the structural integrity of buildings and bridges [41,42,43,44]. Additionally, there may be many other uses for seismology, engineering and Earth sciences, such as array seismology or ray tracing [45].

## 2. Data Acquisition by Multiple-Sensor and Correlation Average Method

Collecting data jointly from multiple sensors and then averaging those data can improve the SNR [45,46]. After calculating the joint average of the data from N sensors, the dynamic range of the network as a whole can be increased by 10 log N (dB), and the self-noise can be attenuated to $\frac{1}{\sqrt{N}}$ for a single sensor [45].

**Figure 2:**
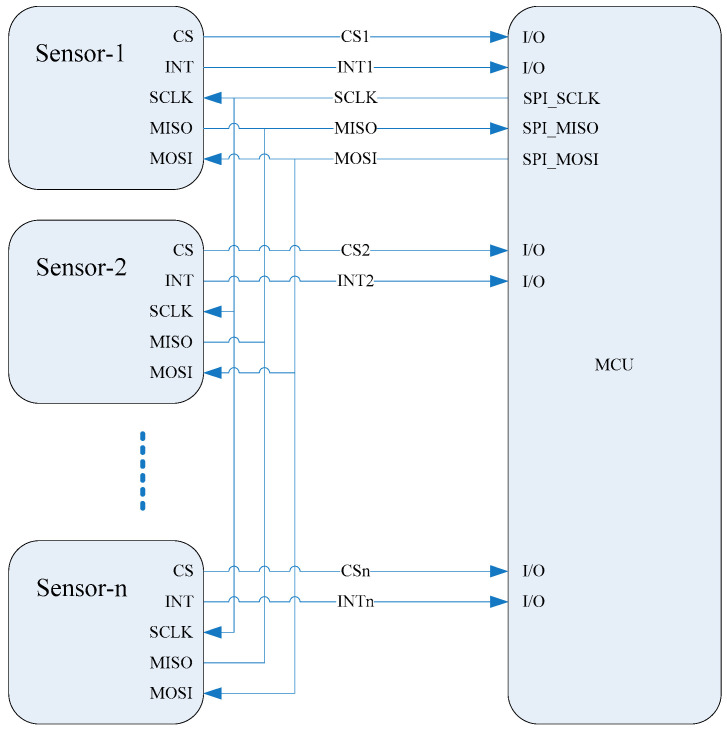
Schematic diagram of the sensors in our instrument, connected in parallel.

**Figure 3:**
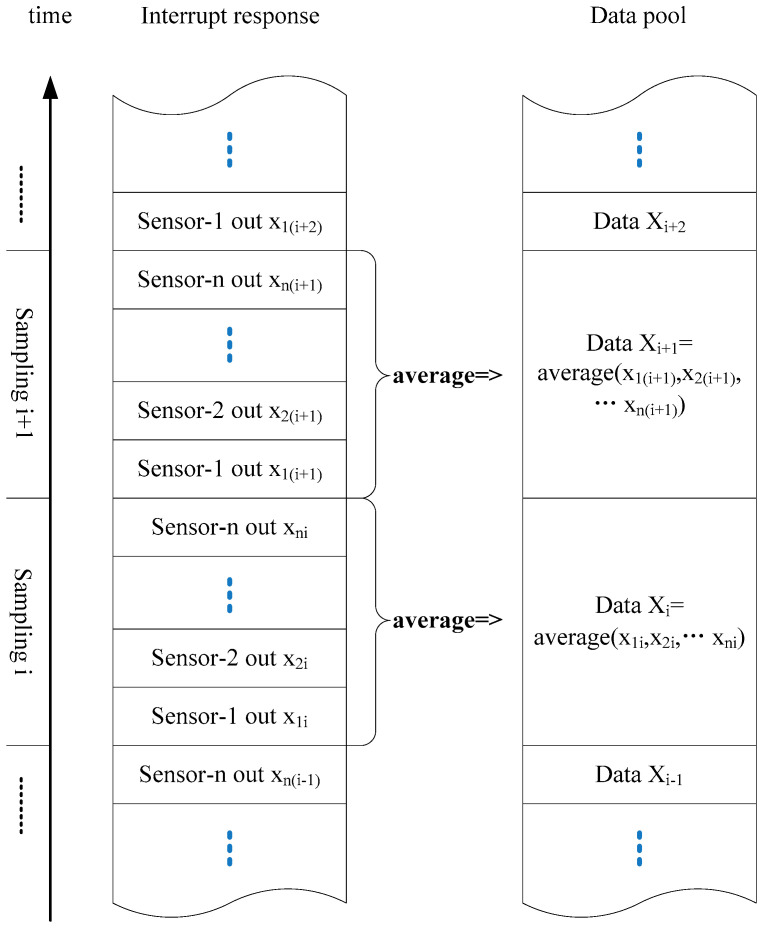
Flowchart representing the data reading and recording process.

Figure 2 shows a schematic diagram of sensors connected in parallel using the serial peripheral interface (SPI). The master out slave in (MOSI), multiple-input single-output (MISO), and simplified clock (SCLK) for all the sensors are public and connected to the SPI of the microprogrammed control unit (MCU) to reduce the number of pins occupied in the MCU. The interrupt signal line (INT) and chip select line (CS) of each sensor are connected to the I/O port of the MCU so that the MCU can send information to and receive information from each sensor. When an interrupt signal occurs, the MCU responds to each interrupt signal sequentially by recording the real-time data via the SPI and storing the data in a public data buffer pool according to the reading order. After reaching a sampling cycle, the recorded data are collected and averaged (Figure 3).

## 3. Instrument Development

### 3.1. System Design

**Figure 4:**
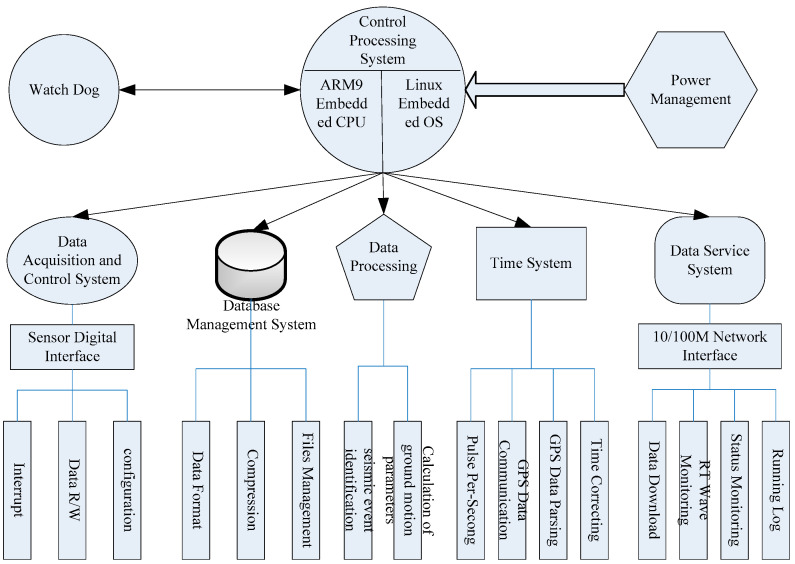
Functional structure of the seismic intensity rapid reporting instrument.

Using a multi-chip, low-cost MEMS digital accelerometer as the seismic sensing unit, a high-performance, low-power 32-bit ARM embedded CPU as the control processor, and Linux as the operating system, we designed a seismic sensor with a low-power data acquisition system. With a built-in large-capacity SD card memory, integrated network communication transmission and control functions, and an embedded real-time seismic data processing algorithm, this instrument is able to automatically calculate ground motion parameters [47]. This small digital microseismograph, which integrates sensors, data acquisition, data storage, and data processing and transmission, consumes less power (less than 2w) and is more affordable (a few hundred USD) to install and maintain than traditional seismometer stations. The seismic sensor is controlled by eight components: the control management module, the data acquisition module, the data storage management module, the data processing function, the GPS time correction function, the data service function, the watchdog function, and the power management function (Figure 4).

The control processing system is used to control and manage the work completed by the other seven components. To optimize the processing power while keeping the cost low, we used a S3C2416 chip (manufactured by Samsung Electronics), a 32-bit low-power ARM9 structured embedded microprocessor, as the central control processor. With its ARM920T core, the S3C2416 chip can integrate the on-chip functions at a maximum operating frequency of 533 MHz. With a running memory of 64 MB, the SDRAM meets the needs of the system operation and data processing software. The 256 MB Nand-Flash main memory serves as the storage location for the system and its applications. To simultaneously manage the different applications, we used a stable 3.1.0 kernel Linux embedded operating system, which also performs tasks related to kernel tailoring, driver design, and startup parameter configuration for each piece of hardware. We employed multi-thread technology to design Linux applications that enable the multi-task parallel processing of various functional modules.

The sensor data acquisition module collects the seismic acceleration signals in real time. In order to increase the dynamic range and reduce the cost of the instrument, multiple low-cost MEMS digital accelerometers (ST’s LIS3DHH) were used for parallel acquisition, and the outputs of all sensors were accumulated and averaged to reduce self-noise.

The data storage system stores the collected data in the SD card. Whenever a strong earthquake occurs, the seismic intensity rapid reporting instrument will immediately acquire the ground motion parameters and transmit them to the monitoring center to provide valuable information for emergency rescue services. The earthquake waveform data and the background noise before the earthquake are also stored for future analysis. Large earthquakes can often damage telecommunications infrastructure, and the surge in communication traffic can lead to a temporary blockage of the network. A large number of sensors on the network simultaneously transmitting real-time seismic waveform data to the monitoring center will exacerbate this network blockage. The instrument lacks a large-capacity local database storage system, so the relevant data can be downloaded after the block has cleared, if needed. The instrument stores three types of data files locally: real-time data (which are stored as a data file per hour), event files, and parameter files containing the ground motion parameters calculated for each event. Real-time data files and event data files are stored in binary format, while ground motion parameter files are stored in text format. Because areas ravaged by large earthquakes may require a long recovery time, it may take one or two months to fully collect or download the recorded data; our instrument can store up to 12 months of data at a sampling rate of 50 SPS in the built-in 32 GB large-capacity SD memory card. Files that exceed the storage are automatically deleted, which frees up the storage space needed to continue to collect new data.

**Figure 5:**
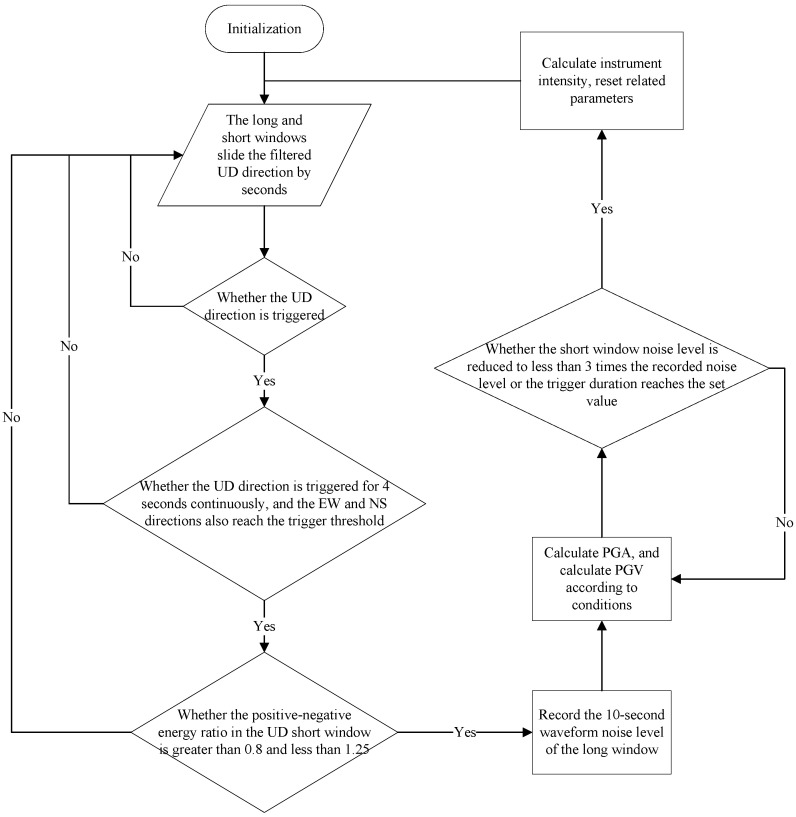
Decision tree representing the seismic event discrimination and calculations needed to determine the ground motion parameters.

The seismic information processing system filters the real-time data, determines the trigger threshold of the seismic events [48,49,50,51], and calculates the PGA and PGV ground motion parameters. Figure 5 shows a flowchart of the seismic event discrimination and calculations required to determine the ground motion parameters.

The GPS time correction system responds to the GPS second interrupt signal, reads GPS information through the serial port, and analyzes the temporal date and the coordinate of latitude and longitude data. When the GPS signal is valid, the successfully read GPS time is used to correct the instrument’s real-time clock to ensure the time accuracy of the data collected by the sensor. If GNSS reception is low, the crystal oscillator with an accuracy of 1 ppm can keep the real-time clock’s accuracy in the short term.

Connected by wired or wireless network, the remote data service system facilitates the status monitoring, real-time waveform monitoring, FTP data file downloading, and parameter setting for the seismic intensity rapid reporting instrument. The watchdog program improves the robustness of the system. By monitoring the running status of the program in real time, the watchdog can restart the program in order to preserve itself in unexpected or disruptive situations.

### 3.2. Acquisition Software

With an embedded Linux operating system that has been tailored to the kernel, as well as a built-in seismic data processing algorithm, our instrument collects ground motion acceleration signals in real time and automatically calculates ground motion parameters. Because the instrument uses Linux as the operating system, it can also run third-party software such as professional extended functions and special algorithms; this flexibility means that this instrument could be useful in a number of other applications.

**Figure 6:**
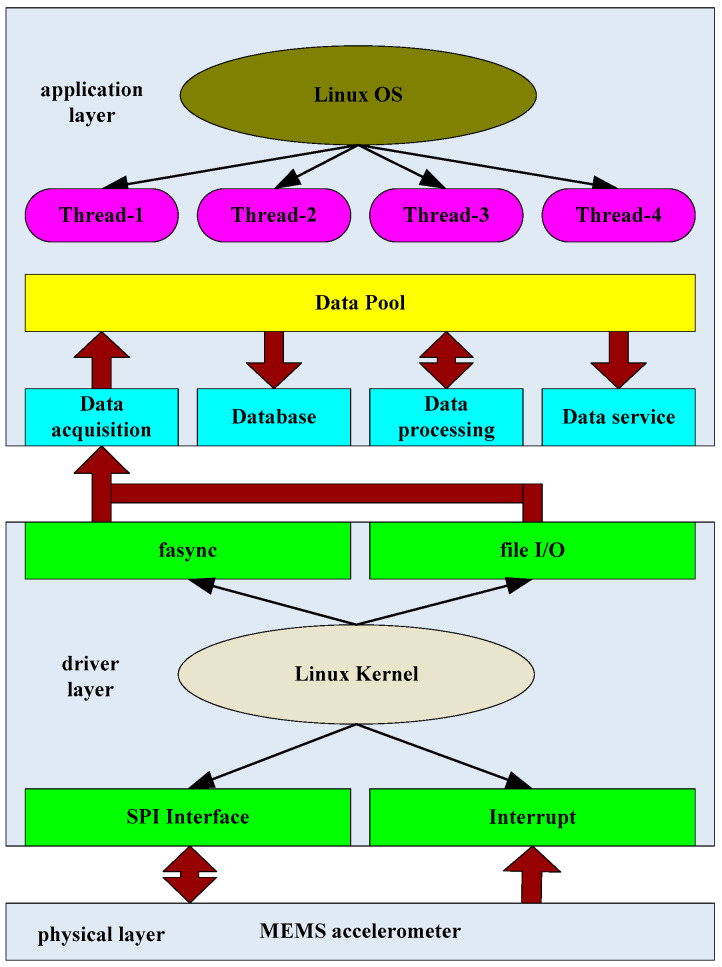
Flowchart representing the data collection process in our software.

The acquisition software consists of layers and modules (Figure 6). Each module is relatively independent, and information exchange between the modules is accomplished via external data interfaces or a shared data buffer pool. The acquisition software consists of an application layer and a driver layer. Using multi-thread technology, the application layer mainly manages the real-time data acquisition module, the local database storage management module, the seismic data processing module, and the remote data service module. Once the real-time data acquisition module receives an asynchronous notification signal from the bottom layer, it wakes up and starts to perform its tasks. The standard file function reads the real-time data from the driver storage space and then stores it in the data buffer pool. The data buffer pool is a storage area in the memory space for periodic reading and writing. The seismic data processing module performs the seismic event discrimination and ground motion parameter calculations using the real-time data stored in the data buffer pool. In addition to creating file directories in which to store and manage real-time data, event waveform data, and parameter data, the local database storage management module also recycles data storage space by deleting data that exceed the time limit. The remote data service module acts as a data remote server and allows clients to continuously receive real-time seismic data and ground motion parameter transmissions, monitor the instrument status, and query and download historical data files. The continuous data transmission adopts the quasi-real-time transmission mode of one frame per second, and the transmission mode of one frame per 0.1 s can be adopted in the application, requiring a smaller delay. A compact frame structure whose length varies only with the sampling rate is adopted. The frame structure mainly composed of frame head constant (4 bytes), acquisition time (8 bytes), latitude and longitude information (10 bytes), sampling rate (4 bytes), data (number of bytes is: 3 channels × 4 bytes × sampling rate), and checksum (4 bytes). At the same time, when composing the network application, the seismic sensor sends heartbeat-data-packets to the monitoring center with another network port number every 5 min to report the network status of the sensor. The heartbeat packet also contains ground motion parameters. When the sensor does not detect seismic events, the transmitted ground motion parameter value is 0. When there is an earthquake, the ground motion parameters are calculated in real time, and heartbeat packets are immediately sent to the monitoring center.

The application in the driver layer configures the sensor, reads sensor data, and implements the file operation functions for reading and writing kernel space data. This application includes the drivers for the hardware data read and write bus interface, the hardware interrupt drivers, the asynchronous notifications for the drivers in the kernel space to send read/write requests to the applications in the user space, and the implementation of file operation functions such as read () and write (). These layered design and modularization methods not only improve the stability and reliability of the software system operation, but also facilitate the upgrading, maintenance, and management of the software.

## 4. Performance Assessment

### 4.1. Self-Noise Test

**Figure 7:**
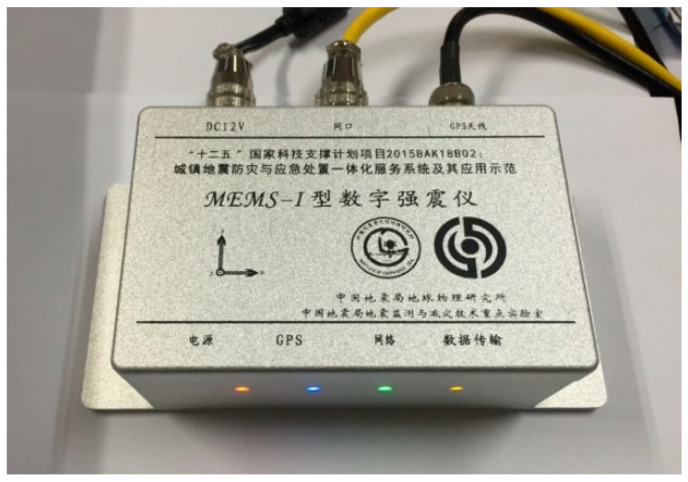
The MEMS seismic intensity rapid reporting instrument developed in this study.

**Figure 8:**
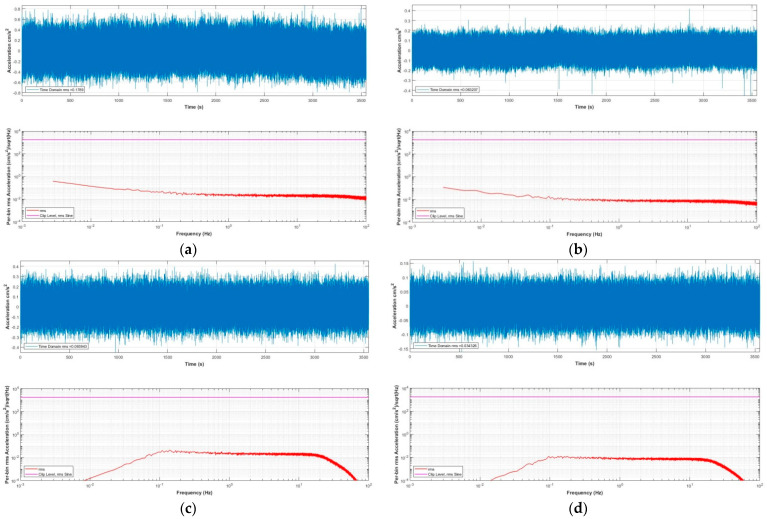
Instrumental noise recording and the spectral density, sampling rate 200 Hz. (**a**) Unfiltered noise, single sensor acquisition; (**b**) unfiltered noise, with eight sensors arranged for parallel acquisition; (**c)** noise with single sensor acquisition, filtered by 0.1–20 Hz bandpass; and (**d**) noise with eight sensors arranged for parallel acquisition, filtered by 0.1–20 Hz bandpass.

Figure 7 is a photograph of our MEMS seismic sensor. The instrument was placed in a quiet basement to continuously record data for one day, and the records during the quiet period at night were interpreted as the instrumental noise. To compare the effectiveness of the single-sensor acquisition method and the multisensor parallel acquisition method, we placed the instrument with a single accelerometer and the instrument with eight accelerometers in parallel close together in the same orientation for simultaneous observation. The recorded noise waves and their corresponding spectra values from the two acquisition methods are shown in Figure 8a–d, respectively. Among them, the built-in matlab function butter() was used to calculate the coefficient of the Butterworth digital filter, and the parameter was set to order 3 with frequency band 0.1–20 Hz. Then, the filter() function was used to filter. In the time domain, the RMS noise (calculated according to sensor data) of the instrument with eight accelerometers in parallel (0.034 cm/s^2^) is noticeably lower than that of the RMS noise for the single-sensor instrument (0.096 cm/s^2^) in the 0.1–20 Hz bandwidth range.

**Figure 9:**
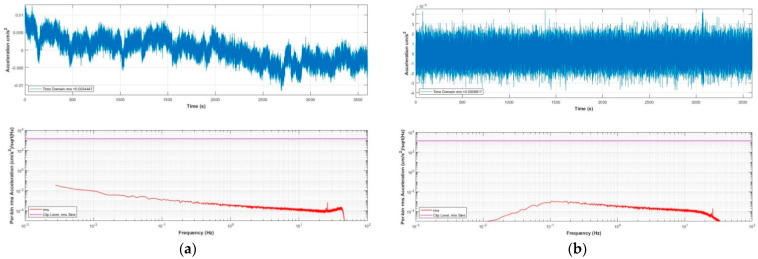
BL-03 force-balance accelerometer recording noise, sampling rate of 100 Hz. (**a**) Unfiltered data; (**b**) data filtered by 0.1–20 Hz bandpass.

A traditional high-precision force-balance accelerometer was used for comparative testing in the same environment. The BL-03 force-balance accelerometer developed by the Institute of Geophysics, China Earthquake Administration was used, with a measurement range of ±2 g and a sensitivity of 4.75 V/g. The recorded results are shown in Figure 9. Figure 9a shows the original data without filtering, and Figure 9b shows the data with 0.1–20 Hz bandpass filtering by using the same filter mentioned above. As can be seen from the figure, the low-frequency noise of the force-balance accelerometer is relatively high due to the influence of its mechanical structure. However, on the whole, its self-noise is much smaller than that of the MEMS seismic sensor developed in this paper. In the bandwidth range of 0.1–20 Hz, its RMS noise is only 0.00088 cm/s^2^. 

### 4.2. Performance Test with a Jolt Table

**Figure 10:**
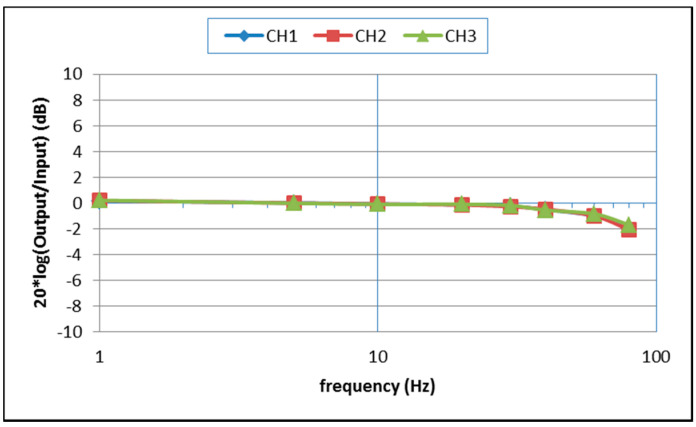
Amplitude–frequency plot of our instrument’s performance on a jolt table.

**Figure 11:**
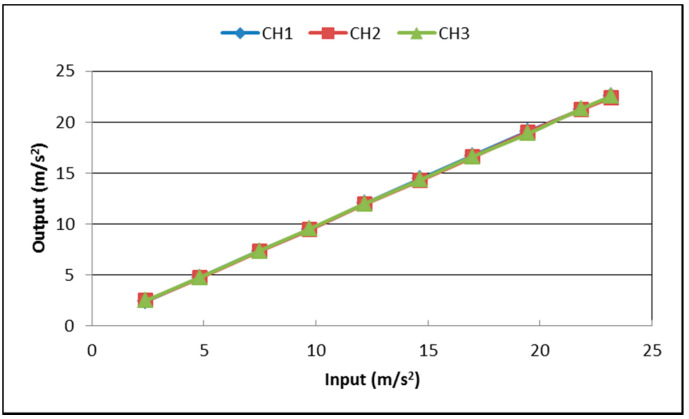
Input and output linear linearity for the data recorded on our instrument when placed on a jolt table.

The frequency response and linearity of the seismic sensor were tested at a jolt table. During the test, the three sensing directions of the seismic sensor were made to coincide with the movement direction of the jolt table separately. The jolt table was set to run a variety of sinusoidal test signals with different amplitudes and frequencies; the results of corresponding amplitude–frequency responses and linearity are shown in Table 1 and Table S1 and Figure 10 and Figure 11. To reduce the need for expensive calibration tools such as long stroke linear shaders [52], we only tested the amplitude–frequency characteristics of the instrument at frequencies higher than 1 Hz. It can be seen that after multiple accelerometer output correlation averagings, the amplitude–frequency response of its input and output within −3 dB is DC −80 Hz (Figure 10), and the linearity is better than 0.47% (Figure 11), less than the maximum nonlinearity in the LIS3DHH manual [53].

### 4.3. Performance Indicators

The main performance indicators of the developed seismic sensor are shown in Table 2, demonstrating that the sensor can meet the network access standard of the China Earthquake Administration’s high-precision seismic intensity meter (Table 2) [54].

## 5. Experimental Seismic Monitoring Network

**Figure 12:**
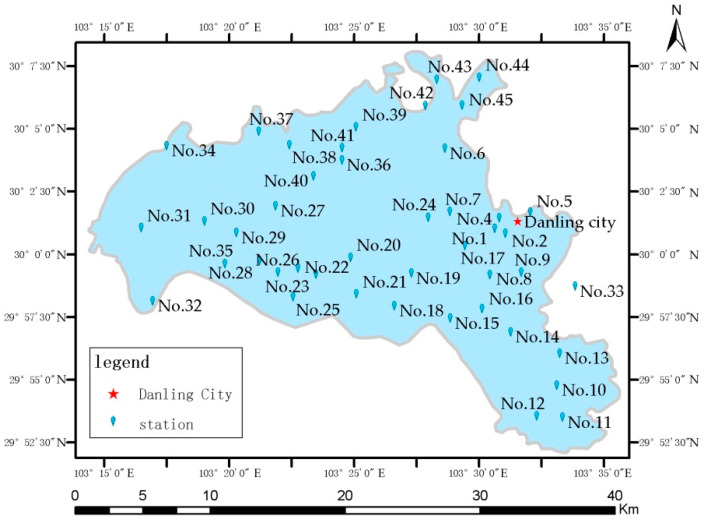
Map of the experimental seismic monitoring network in Sichuan, China.

**Figure 13:**
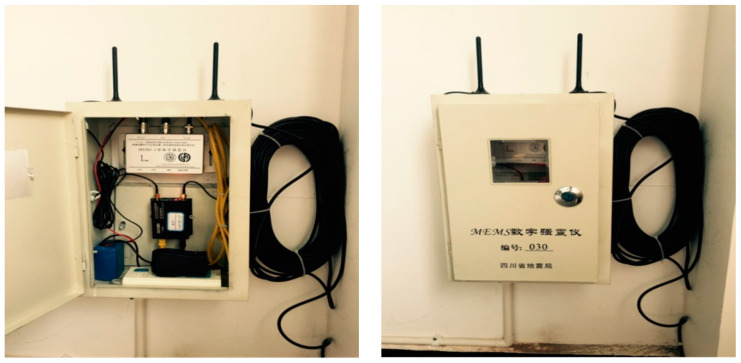
The installation of the seismic sensor in a village hall in Sichuan.

The seismic sensor has been applied in several projects since its development. Figure 12 shows an experimental seismic monitoring network composed of 45 sensors in Sichuan, a province in China where earthquakes occur frequently. The monitoring system consists of the seismic sensor network, the communication network, and the data collection and network monitoring center. To reduce the cost of monitoring, we installed the seismic sensor on the interior wall of the village committee hall, an area with low noise. The instrument was mounted at a height of 0.6 m from ground level; the accessibility of the instrument reduced construction and maintenance costs. The seismic sensor, lithium battery, power adapter/battery charger, power socket, and 3G/4G wireless router were all stored in a small iron electric meter box for protection (Figure 13); the sensor was tightly coupled to the wall and fixed with set screws. Except for the 4G wireless router, which costs a few dozen USD, other materials cost only a few USD. Therefore, excluding labor, the cost of a complete unit is less than USD 1000.

Because the instruments were installed in very different areas, it was not possible for each instrument to be hardwired into a network. In those cases, we used low-cost 3G/4G wireless network routers to transmit the recorded data. With a L2TP tunnel protocol connecting the instrument to the virtual private network (VPN) of the seismic system, users could use the low-cost VPN intranet to remotely monitor the real-time seismic waveform, download data using FTP, and update and maintain the system software.

**Figure 14:**
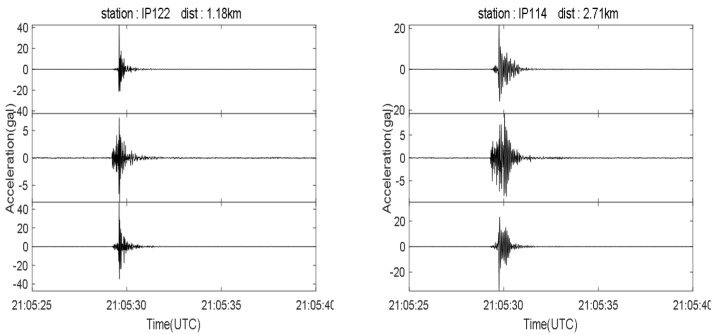

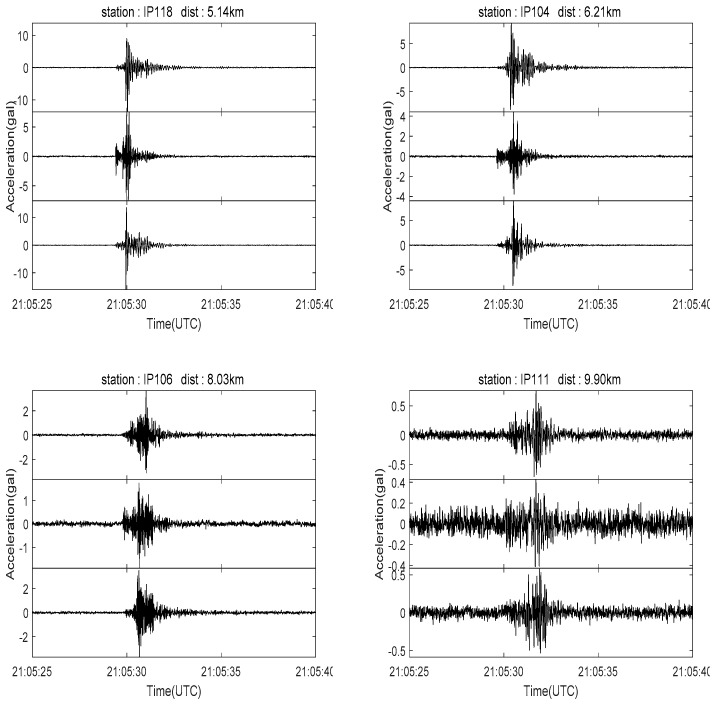
Seismogram from a close range M2.2 earthquake that occurred at 21:05:27 (UTC time), 23 July 2020, with a hypocentral depth of 3 km, a sampling rate of 200 Hz, and a bandpass filter of 0.1–20 Hz.

**Figure 15:**
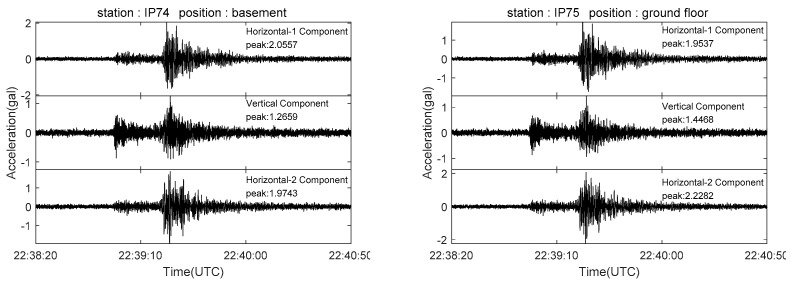

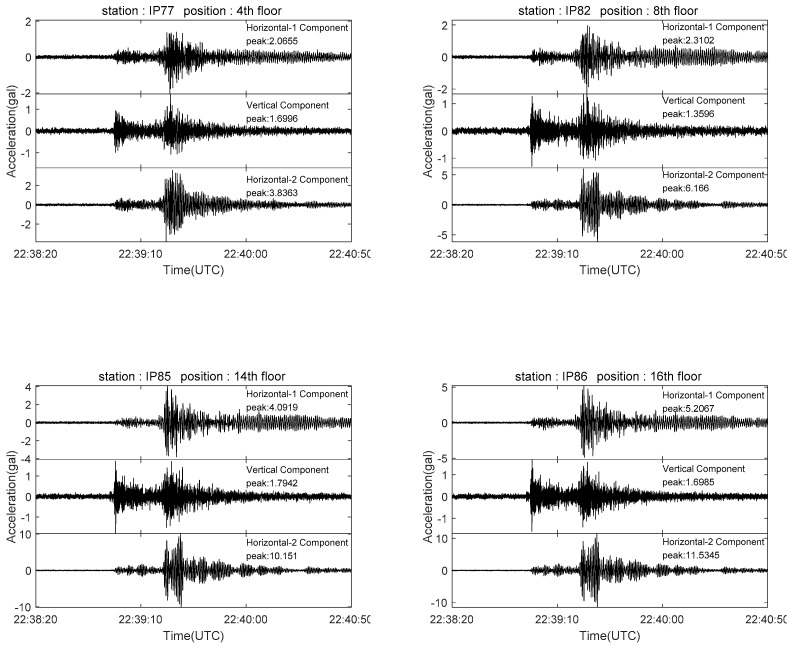
Seismograms from the Tangshan M5.1 earthquake that occurred at UTC 22:38:00, 11 July 2020. These seismograms were recorded on different floors of Beijing’s Building Monitoring Network. The hypocentral depth of the earthquake was 10 km. The building was 191 km away from the epicenter. The sensor’s sampling rate was 200 Hz. Additionally, the data were filtered by a bandpass filter of 0.1–20 Hz (the seismogram data for this study were provided by Beijing Earthquake Agency).

Our monitoring network recorded multiple earthquakes; the recorded seismic phases are clear and highly distinctive. Figure 14 shows an M2.2 close-range earthquake recorded by stations at different distances. Another sensor network was set up located near Beijing, mounted on a 17-story building to monitor the structural impact of earthquakes. Figure 15 shows the Tangshan M5.1 earthquake waveform recorded on 12 July 2020 by the network, 191 km away from the epicenter [55].

## 6. Discussion

The low-cost MEMS seismic sensor consists of MEMS digital accelerometers, and a digital processing system that realizes functions such as control, storage, communication, and time calibration. When the cost of the seismic sensor is much greater than the cost of the MEMS digital accelerometer used (for example, the total cost of the seismic-sensor is a few hundred USD and the single accelerometer sensor costs a few USD, for which the cost ratio is approximately 100:1), the cost effectiveness of the instrument is greatly improved using the correlation average method of multiple MEMS digital accelerometers. If eight MEMS accelerometers are used, the resolution of the sensor can be increased by 2.8-fold, while the increased cost is less than 10% of the overall cost. However, if a high-precision MEMS accelerometer is used, the cost of the sensor is mainly the cost of the MEMS accelerometer. In such a scenario, the method of using multiple MEMS accelerometers in parallel can only improve the resolution of the instrument, while the cost performance is not improved.

## 7. Conclusions

In this paper, eight LIS3DHH digital MEMS accelerometers are used to develop a MEMS seismic sensor with high cost performance by using an ARM embedded controller through the correlation average acquisition method. The test results show that the RMS noise of the instrument is reduced from 0.096 cm/s^2^ with a single MEMS accelerometer to 0.034 cm/s^2^ in a bandwidth of 0.1–20 Hz by using the correlation average method of eight low-cost MEMS accelerometers. The dynamic range reaches more than 90 dB, the amplitude–frequency response of its input and output within −3 dB is DC −80 Hz, and the linearity is better than 0.47%. With the characteristics of small size, low cost, low power consumption, integration and intelligence, it can be applied to a variety of seismic monitoring applications. At the same time, the seismic sensor is easy to install in practical application, which can make the cost of building a station less than USD 1000, and can greatly reduce the cost of monitoring. In earthquake monitoring projects, such as earthquake intensity rapid reporting and earthquake early warning, the cost and performance of seismic sensors affect the installation density and monitoring capabilities of the earthquake monitoring network, which, in turn, affect the performance of the network system. Seismic sensors using multiple low-cost MEMS digital accelerometers can greatly improve the monitoring capabilities of the sensors on the basis of only a small increase in cost, and have a high cost performance. Therefore, the approach proposed in this paper is particularly suitable for applications that require the high-density deployment of instruments, such as earthquake intensity rapid reporting and earthquake early warning networks.

## Figures and Tables

**Table 1:** Amplitude–frequency data from our instrument recorded during a session on a jolt table (sampling rate: 200 SPS).

**Test frequency (Hz)**		1	5	10	20	30	40	60	80
**Peak input value of the jolt table (m/s^2^)**		4.03	7.05	7.14	7.07	7.07	7.07	7.07	7.07
**CH1**	**Sensor output(m/s^2^)**	4.11	7.07	7.09	6.97	6.88	6.67	6.31	5.67
	**20log (output/input) (dB)**	0.17	0.02	−0.06	−0.13	−0.23	−0.51	−0.99	−1.91
**CH2**	**Sensor output (m/s^2^)**	4.12	7.05	7.08	6.95	6.85	6.70	6.30	5.57
	**20log (output/input) (dB)**	0.20	0.00	−0.07	−0.14	−0.28	−0.47	−1.00	−2.08
**CH3**	**Sensor output (m/s^2^)**	4.13	7.04	7.07	7.00	6.92	6.64	6.42	5.81
	**20log (output/input)** **(dB)**	0.22	−0.01	−0.09	−0.09	−0.18	−0.54	−0.84	−1.70

**Table 2:** Correlation average seismic sensor development index and China Earthquake Administration’s high-precision seismic intensity calculation standard.

Technical Indicators or Functional Indicators	Technical Requirements of China Earthquake Administration’s Seismic Intensity Meter into the Network	Indicators Reached by the Developed Instrument
**Number of channels**	3	3
**Sampling rate**	50 SPS, 100 SPS, 200 SPS	50 SPS, 100 SPS, 200 SPS
**Full scale measurement range**	±2 g (−19.6 m/s^2^–19.6 m/s^2^)	±2.5 g (−24.5 m/s^2^~24.5 m/s^2^)
**Self-noise RMS**	0.1 mg @0.1–20 Hz	About 0.03 mg @0.1–20 Hz
**Linearity**	Better than 1%	Better than 0.47%
**Measuring error**	Less than 5% (0.1–20 Hz)	Better than 3.4% @10 Hz
**Frequency response**	Low cut-off frequency: ≤0.01 Hz High cut-off frequency: ≥40 Hz (−3 dB, at a sampling rate of 100 Hz or 200 Hz) High cut-off frequency: ≥20 Hz (−3 dB, at a sampling rate of 50 Hz)	DC ~80 Hz (@200 SPS)
**Dynamic range**	>60 dB (0.1–20 Hz, when the observed data is used only for seismic intensity measurement) >80 dB (0.1–20 Hz, when the observed data is used for both seismic intensity measurement and earthquake early warning)	>90 dB (0.1–20 Hz)
**GPS time correction**	Equipped with the function of GNSS time correction	
**Monitoring of real-time data waveform**	Equipped with the function of monitoring real-time data waveform	
**Downloading of remote data file FTP**	Equipped with the function of downloading remote data file FTP	
**Continuous waveform file storage**	Equipped with the function of storing continuous waveform files	
**Event waveform file storage**	Equipped with the function of storing event waveform files	

## Data Availability

Generated during the study.

## Supplementary Materials

The following are available online at https://www.mdpi.com/article/10.3390/s21237970/s1, Table S1, Linear characteristic test (test frequency 10 Hz).
