# Supplementary material for: Improved Resolution and Cost Performance of Low-Cost MEMS Seismic Sensor through Parallel Acquisition
Source: Sensors (Basel). 2021 Nov 29;21(23):7970. doi: 10.3390/s21237970 (PMC8659451; doi:10.3390/s21237970)
Supplement: Supplementary file 1 [file sensors-21-07970-s001.zip › sensors-1457972-supplementary.pdf]

sensors

MDPI

Supplementary

Table S1. Linear characteristic test (test frequency 10 Hz)

Jolt table input (m/s²)		2.41	4.83	7.48	9.72	12.19	14.65	17.00	19.48	21.85	23.19
Sensor output (m/s²)	CH1 output	2.41	4.77	7.36	9.46	12.01	14.42	16.72	19.11	21.25	22.59
	CH1 measurement error	0.0%	-1.2%	-1.6%	-2.7%	-1.5%	-1.7%	-1.6%	-1.9%	-2.7%	-2.6%
	CH 1 linearity	0.40%									
	CH2 output	2.47	4.71	7.32	9.45	11.96	14.26	16.57	19.03	21.24	22.41
	CH2 measurement error	2.5%	-2.5%	-2.1%	-2.8%	-1.9%	-2.7%	-2.5%	-2.3%	-2.8%	-3.4%
	CH2 linearity	0.47%									
	CH3 output	2.48	4.77	7.37	9.53	12.00	14.33	16.63	18.88	21.32	22.54
	CH3 measurement error	2.9%	-1.2%	-1.5%	-2.0%	-1.6%	-2.2%	-2.2%	-3.1%	-2.4%	-2.8%
	CH 3 linearity	0.40%									
